# Clinical study to monitor dentinal hypersensitivity with episodic use of a desensitising dentifrice

**DOI:** 10.1038/bdjopen.2017.11

**Published:** 2017-06-23

**Authors:** Stephen Mason, Rose Kingston, Lucy Shneyer, Máiréad Harding

**Affiliations:** 1 Oral Care Medical Affairs, Research and Development, GSK Consumer Healthcare, Weybridge, Surrey, UK; 2 Oral Health Services Research Centre, Cork University Dental School and Hospital, Wilton, Cork, Ireland; 3 Shneyer Statistics LLC, Denville, NJ, USA

## Abstract

**Objectives/Aims:**

To evaluate continuous and episodic twice-daily usage regimens of a desensitising dentifrice containing 5% calcium sodium phosphosilicate (CSPS).

**Materials and Methods:**

In this exploratory, single-centre, randomised, examiner-blind study, subjects with dentinal hypersensitivity were randomised to continuous (24 weeks) use of a 5% CSPS-containing dentifrice or episodic use of the dentifrice comprising two 8-week treatment periods separated by 8 weeks′ use of a standard fluoride dentifrice. Sensitivity was assessed by tactile threshold (Yeaple probe) and evaporative (air) sensitivity (Schiff sensitivity score). Other measures included labelled magnitude scales to assess subjects′ responses to the evaporative stimulus, the Dentine Hypersensitivity Experience Questionnaire and a tooth sensitivity question.

**Results:**

Seventy-six subjects were randomised to continuous (*n*=38) or episodic (*n*=38) use. Small but statistically significant improvements from baseline in Schiff sensitivity scores were observed at weeks 8, 16 and 24 with both regimens (all *P*<0.05). Increases from baseline in tactile threshold were not statistically significant. No significant between-regimen difference was observed for any endpoint. No treatment-related adverse events were reported.

**Discussion:**

Dentifrice containing 5% CSPS improved dentinal hypersensitivity with both episodic and continuous twice-daily usage regimens over 24 weeks and was well tolerated.

**Conclusion:**

No performance differences were observed between the two usage regimens.

## Introduction

Dentinal hypersensitivity is a common oral condition characterised by pain derived from exposed dentine in response to chemical, thermal, tactile or osmotic stimuli, which cannot be accounted for by any other dental defect or disease.^1,2,3^ Hypersensitivity develops as a result of gingival recession, and/or erosion and abrasion of enamel, leading to exposure of the underlying dentine.^4^ The hydrodynamic theory of dentinal hypersensitivity hypothesises that when a stimulus is applied to dentine, the movement of fluid within exposed patent dentinal tubules stimulates the nerve processes in the pulpal area of the dentine to transmit a signal that is perceived as pain.^5^

Treatments for dentinal hypersensitivity are generally based on one of two approaches—the use of depolarising agents, such as potassium ions, with the aim of blocking neural transmission of the pain stimulus, or the use of tubule-occluding agents that physically block exposed dentinal tubules. These blocking agents include strontium, oxalate or stannous salts; arginine; bioglasses; and silicas, which serve to seal the dentine tubules, thereby reducing dentinal-fluid movement in response to external stimuli.^6,7,8, 9,10,11^ Calcium sodium phosphosilicate (CSPS; Novamin, GSK Consumer Healthcare, Weybridge, UK) is a particulate, bioactive material incorporated into oral healthcare products indicated for the treatment of dentinal hypersensitivity. When CSPS particles come into contact with an aqueous environment, such as saliva, there is an immediate release of sodium ions, leading to a localised pH increase due to cation exchange. Together with a release of calcium and phosphate ions, this facilitates the precipitation of an occlusive calcium phosphate hydroxycarbonate apatite-like layer over the exposed dentine.^7, 12,13,14, 15^ The efficacy of dentifrices containing 5% CSPS in reducing dentinal hypersensitivity has been demonstrated in randomised controlled clinical studies of up to 8 weeks′ duration.^16,17,18,19,20,21,22,23,24,25^ A reduction in sensitivity is generally reported following 2–4 weeks′ treatment with 5% CSPS, with further improvements observed with continued twice-daily brushing. *In vitro* studies of CSPS-containing dentifrices have demonstrated maintenance of the occlusive layer following exposure to dietary acid.^26^

Currently there is no available evidence-based information on which the dental healthcare professional can base advice regarding continuous versus episodic long-term approaches to daily use of a desensitising dentifrice for the management of dentinal hypersensitivity. A number of clinical studies have shown that when use of a desensitising dentifrice is discontinued, the pain relief achieved during treatment is gradually lost and sensitivity begins to return.^23,27,28^ For example, one study has reported a degree of recurrence of sensitivity pain within 3 weeks of discontinuation of a dentifrice containing 5% CSPS.^23^ Given the episodic nature of dentinal hypersensitivity and the effectiveness of treatment, it is likely that individuals with the condition will use desensitising products intermittently, depending on the resolution and recurrence of their symptoms. However, in general, the design of clinical studies investigating desensitising agents has not reflected this real-world consumer behaviour. Insights into the consequences of intermittent use can be provided by incorporation into the clinical study design of a transient treatment-withdrawal or ‘regression’ phase, during which evaluation of treatment outcomes continues following cessation of active treatment.

This exploratory study was designed to compare dentine hypersensitivity over a 24-week period of either episodic or continuous use of a desensitising dentifrice containing 5% CSPS and 1,426 p.p.m. fluoride (as sodium monofluorophosphate (SMFP)) as measured by Schiff sensitivity score and tactile threshold (Yeaple probe). The episodic-use regimen comprised two 8-week treatment periods separated by an 8-week non-treatment period (use of a standard fluoride dentifrice). Other exploratory objectives were: to monitor dentine hypersensitivity using labelled magnitude scales (LMSs), the Dentine Hypersensitivity Experience Questionnaire (DHEQ) and a tooth sensitivity question (TSQ); to investigate the relationship between frequency of dietary ‘acidic challenge’ and dentinal hypersensitivity; and to monitor oral tolerability.

## Materials and Methods

### Study design

This was an exploratory, 24-week, single-centre, randomised, examiner-blind, two-treatment arm, parallel-group study in healthy adult volunteers with self-reported and clinically diagnosed dentinal hypersensitivity. The study was conducted at the Oral Health Services Research Centre, Cork, Ireland. The protocol was approved by an independent ethics committee (Clinical Research Ethics Committee of Cork University Teaching Hospitals) and the study was carried out in accordance with the requirements of the Declaration of Helsinki. There was one minor protocol amendment to clarify the meaning of ‘study site’ as stated in the exclusion criteria.

All subjects were required to provide written informed consent before participating in the study. Eligible subjects completed study visits at screening, baseline (⩾7 days post screening), and after 2, 4, 8, 10, 12, 16, 18, 20 and 24 weeks of study treatment. At the screening visit, subjects’ demographics and medical history were recorded and an oral soft tissue (OST) examination was conducted. Each subject’s dentition was then assessed sequentially for: evidence of erosion, abrasion and facial/cervical gingival recession; gingival health status; tooth mobility; and sensitivity to an air-blast stimulus (where a ‘yes’ response from the subject when questioned indicated sensitivity). To provide a standardised oral hygiene regimen before the start of the treatment period, eligible subjects were supplied with a standard fluoride dentifrice (Colgate Cavity Protection, containing 1,000 p.p.m. fluoride as SMFP and 450 p.p.m. fluoride as NaF; Colgate-Palmolive UK, Guildford, UK) and a toothbrush (Aquafresh Clean Control; GSK Consumer Healthcare, Weybridge, UK) for twice-daily brushing (1 min in the morning and evening) for at least 1 week between screening and the baseline visit. Brushing with the lead-in dentifrice was supervised on first use at the study site and was recorded thereafter by subjects in a daily diary.

At the baseline visit, subjects were assessed for ongoing eligibility, their compliance with the lead-in dentifrice was monitored and an OST assessment was conducted. The sensitivity of the eligible teeth identified at screening was assessed using a tactile stimulus (Yeaple probe).^29^ Teeth with a tactile threshold ⩽20 g were then evaluated for sensitivity to an evaporative (air) stimulus (using the Schiff Sensitivity Scale^30^ and LMSs).^31,32^ Based on the Schiff sensitivity score, the dental examiner selected two eligible test teeth to be evaluated for the rest of the study. Subjects were then randomised (1:1) to a continuous or episodic usage regimen of the study dentifrice, which contained 5% (w/w) CSPS and 1,426 p.p.m. fluoride as SMFP (Sensodyne Repair and Protect; GSK Consumer Healthcare, Weybridge, UK).

Randomisation was stratified by maximum baseline Schiff sensitivity score (either 2 or 3) of the two selected test teeth according to a randomisation schedule generated by the Biostatistics Department of GSK Consumer Healthcare. Subjects within each stratum were sequentially assigned a randomisation number in ascending order. The dental examiner, study statistician, data management staff and employees of the sponsor who might influence the study outcomes were blinded to treatment allocations. The study dentifrice and the lead-in dentifrice were supplied in commercial tubes; the study dentifrices were overwrapped to mask their identity as far as possible. Maintenance of the blind was confirmed by inspection of supplied products returned after each 8-week period of the study and by checking that the emergency-use randomisation list had not been accessed.

Subjects were instructed to apply the study dentifrices with the supplied standard manual toothbrush for 1 min twice daily (morning and evening). Those randomised to the continuous-regimen group used the 5% CSPS dentifrice over a continuous 24-week period. Subjects randomised to episodic treatment used the same dentifrice over two 8-week treatment periods separated by an 8-week phase during which they used the standard fluoride dentifrice (Colgate Cavity Protection), which has no known desensitising efficacy. First use of the study treatment was supervised at the study site. Subjects in both groups received new dentifrice and toothbrushes at the start of each 8-week treatment phase. Subjects’ compliance with the administration of the dentifrices was assessed by review of subject-completed diaries at each study visit.

The sensitivity of the two test teeth selected at baseline was re-assessed in response to a tactile stimulus (tactile threshold) and evaporative (air) stimulus (Schiff sensitivity score and LMSs) by the same dental examiner for each measure (one examiner per measure) at weeks 2, 4, 8, 10, 12, 16, 18, 20 and 24. Subjects underwent an OST examination at each visit, before the clinical assessments of sensitivity. Supervised brushing was carried out at each visit to facilitate compliance.

Other evaluations included the DHEQ, completed by subjects at baseline and 8, 16 and 24 weeks; subjects’ weekly responses to the TSQ; and subjects’ estimates of the number of dietary acidic challenges per day.

During the study, subjects were not permitted to use any oral-care products other than those provided or any dental products (including home remedies) intended for treating tooth sensitivity. Subjects were required to abstain from use of interdental cleaning aids (except to remove impacted food) and to avoid any non-emergency dental treatment, including prophylaxis. Subjects were requested to refrain from excessive alcohol consumption for 24 h before each visit, from all oral hygiene procedures and use of chewing gum for at least 8 h, and from eating, drinking and smoking for at least 4 h.

### Subjects

Eligible subjects were aged 18–50 years and in good general health with pre-existing (⩾6 months and ⩽10 years), self-reported and clinically diagnosed dentinal hypersensitivity. At screening, subjects were required to have at least 20 natural teeth, including at least four accessible non-adjacent teeth (incisors, canines or premolars) that met all of the following criteria: evidence of erosion, abrasion and facial/cervical gingival recession; a Gingival Index (GI) score ⩽1; a clinical mobility score ⩽1; and sensitivity to an air-blast stimulus. At baseline, subjects eligible for randomisation were required to have at least two accessible, non-adjacent teeth (incisors, canines or premolars) demonstrating signs of sensitivity as determined by a tactile threshold ⩽20 g and a Schiff sensitivity score ⩾2.

General exclusion criteria included pregnancy; breastfeeding; intolerance or hypersensitivity to the study dentifrices or their ingredients; participation in a clinical study or receipt of an investigational drug within 30 days of screening; participation in a tooth-desensitising study within 8 weeks of screening; use of sensitivity oral care products within the previous 8 weeks; presence of any chronic debilitating disease that could influence study outcomes; any condition causing clinically relevant xerostomia; daily use of any medications that might influence the perception of pain or cause xerostomia; and a requirement for antibiotic prophylaxis before dental treatment.

General dentition exclusion criteria were: dental prophylaxis within 4 weeks of screening; tongue or lip piercing; desensitising treatment or tooth bleaching within 8 weeks of screening; gross periodontal disease; treatment of periodontal disease (including surgery) within 12 months of screening; scaling or root planing within 3 months of screening; active caries or periodontitis; and partial dentures, orthodontic appliances, implants or restorations in a poor state of repair that could influence study outcomes. Specific exclusions for the two selected test teeth were: evidence of current/recurrent caries or decay in the previous 12 months; exposed dentine but with deep, defective or facial restorations; teeth used as abutments for fixed or removable partial dentures; teeth with full crowns or veneers, orthodontic bands or cracked enamel; and sensitive teeth with contributing aetiologies other than erosion, abrasion and facial/cervical gingival recession or considered by the investigator as unlikely to respond to an over-the-counter dentifrice.

### Assessments

At screening, eligibility assessments included evaluation of gingival health using the four-point (0–3) GI scale.^33^ For teeth with a GI score ⩽1, tooth mobility was scored from 0 to 3 using a modification to the Miller index^34^ as follows: 0=no movement or mobility of the crown of the tooth <0.2 mm in a horizontal direction; 1=mobility 0.2–1.0 mm in a horizontal direction; 2=mobility >1 mm in a horizontal direction; 3=mobility in a vertical direction as well as in a horizontal direction.

At each post-screening visit, as recommended by consensus guidelines,^35^ two independent stimulus-based efficacy measures were used to assess dentinal hypersensitivity: tactile sensitivity was assessed for the two designated test teeth, followed by an evaporative (air) sensitivity test with a minimum of 5 min between tests. Each measure was assessed by a single, different examiner for the duration of the study. Examiners were already experienced in the use of these assessments and were also expected to undergo calibration, refresher training and re-familiarisation with the techniques, as necessary. Tactile sensitivity was measured by applying a constant-pressure Yeaple probe^29^ that was calibrated on each day it was used. Testing was initiated at 10 g and increased in increments of 10 g until either two consecutive ‘yes’ responses (with ‘yes’ indicating that the stimulus caused pain or discomfort) were elicited from the subject at the same pressure setting (which was recorded as the tactile threshold in grams) or the maximum force was reached. At baseline, the maximum force was set at 20 g; at subsequent visits it was 80 g. The greater the tactile threshold (i.e., the greater the pressure the subject was able to tolerate), the less sensitive the tooth.

The evaporative (air) sensitivity test was assessed by application of a 1-s blast of air from a triple air dental syringe to the exposed dentine surface of the isolated test tooth. The subject’s response was recorded by the examiner on the four-point Schiff Sensitivity Scale as: 0=no response; 1=subject responds to air stimulus but does not require withdrawal of stimulus; 2=subject responds to air stimulus and requests withdrawal of stimulus or moves from stimulus; 3=subject responds to air stimulus, considers stimulus to be painful, and requests discontinuation of stimulus.^30^ In addition, subjects used the LMSs immediately after the evaporative (air) stimulus to rate the intensity, duration, tolerability and descriptive quality of their response to the stimulus.^31,32^ Training in the use of the LMSs was given at baseline, and weeks 8, 16 and 24.

Before OST examination and tooth sensitivity assessments were conducted, subjects also completed the 48-item DHEQ, a validated condition-specific questionnaire used to assess the impact of dentinal hypersensitivity on oral health-related quality of life.^36,37^ The questionnaire assesses an individual’s experience of dentinal hypersensitivity in terms of sensation, their impression of the impact of the condition on various aspects of daily life and their global oral health.

Subjects also used the TSQ to score the sensitivity of their teeth on a scale of 0 (no discomfort) to 3 (severe pain in response to things that usually cause sensitivity). In addition, subjects recorded the number of daily dietary ‘acidic challenges’ (i.e., the number of times they consumed an acidic food or beverage that day), based on provided examples of typical acidic challenges.

### Safety

Adverse events (AEs) and OST abnormalities were monitored at each study visit. AEs were recorded from the first use of the acclimatisation dentifrice (at the screening visit) until 5 days after the last use of study treatment. Any relationship between study treatment and the occurrence of an AE was assessed by the investigators, who also graded the intensity of the AE as mild, moderate or severe.

### Data analyses

As the study was exploratory and was not powered to detect any treatment differences, no formal sample-size calculations were performed. Sufficient numbers of subjects were screened to allow ~35 subjects per randomised group.

The intent-to-treat (ITT; primary analysis) population comprised all randomised subjects who received study treatment at least once and had at least one post-baseline assessment of efficacy. An analysis of the per-protocol population (i.e., all subjects in the ITT population who had at least one assessment of efficacy considered unaffected by protocol violations) was not performed as <10% of data were excluded from the per-protocol population. Treatment-emergent AEs were reported for the safety population, which included all randomised subjects who received at least one administration of study treatment.

Mean Schiff sensitivity score, tactile threshold and LMS scores, and changes from baseline were calculated across the two test teeth at each timepoint for each subject. Changes from baseline at weeks 8, 16 and 24 were analysed using a repeated-measures analysis of covariance model with fixed effects for treatment, visit, treatment by visit interaction and baseline Schiff sensitivity score stratification (except for the model for Schiff sensitivity score analysis), and with baseline Schiff sensitivity scores, tactile threshold or LMS score as a covariate, dependent on the variable being analysed. Subject was included as a random effect. Assumptions of normality were investigated for all endpoints. Tactile threshold data were found to violate that assumption, therefore for this endpoint median differences are presented with 95% confidence intervals (CIs) based on the Hodges–Lehmann method and *P* values for between-regimen comparisons were based on the Wilcoxon rank-sum test.

Additional measures were analysed as unadjusted means (±s.e.) for single DHEQ questions, DHEQ subscale and composite scores, and total score; medians for TSQ scores and number of subjects experiencing total relief (post-treatment response of 0=no discomfort or awareness of sensitivity); number of improvers (post-treatment improvement in response); and means for number of acidic challenges per day. Full data for these endpoints will not be presented.

## Results

### Subjects

**Figure 1:**
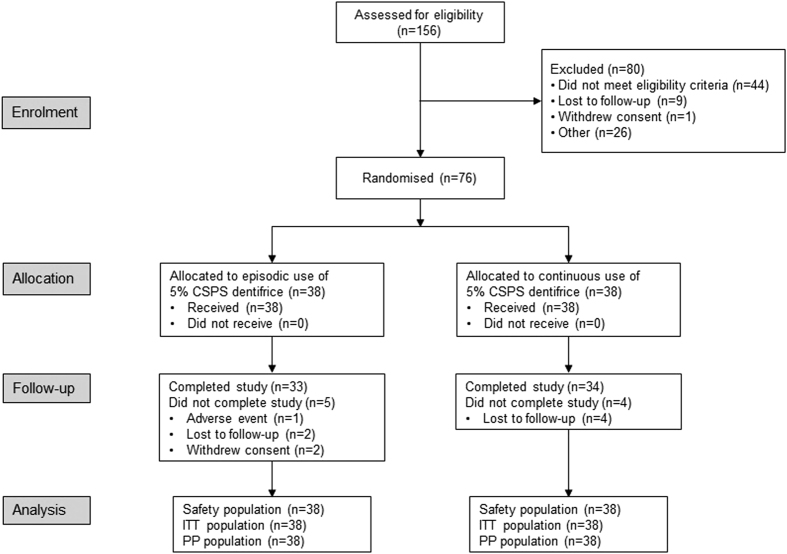
Subject disposition. CSPS, calcium sodium phosphosilicate; ITT, intent-to-treat; PP, per protocol.

The first subject was enroled on 29 October 2013 and the last subject completed the study on 2 May 2014. A total of 156 subjects were screened and 76 were randomised to continuous (*n*=38) or episodic use (*n*=38) of the study dentifrice and were included in the ITT and safety populations (Figure 1). Characteristics were comparable between the two groups (Table 1). There was a higher proportion of female versus male subjects: 57.9 versus 42.1% overall. The mean age of the subjects was 29.8 (s.d. 10.29; range 18–48) years and almost all (98.7%) were White. Similar proportions of subjects in each of the strata were defined by maximum baseline Schiff sensitivity score of 2 or 3.

### Efficacy

**Figure 2:**
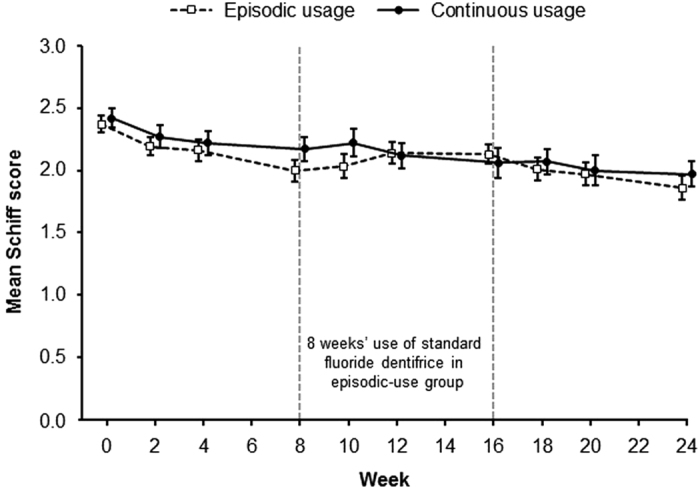
Mean (±s.e.) Schiff sensitivity scores during continuous and episodic use of a desensitising dentifrice containing 5% calcium sodium phosphosilicate (intent-to-treat population). Data have been offset for clarity.

The mean Schiff sensitivity scores (±s.e.) for the episodic- and continuous-use groups over the study duration are shown in Figure 2. The two treatment groups showed similar profiles over the 24-week study period, with small but statistically significant (*P*<0.05) decreases from baseline, indicating an improvement in sensitivity (see Table 2 for adjusted mean change from baseline including 95% CIs). No statistically significant differences between continuous and episodic use were observed for change from baseline at weeks 8, 16 or 24 (Table 2).

**Figure 3:**
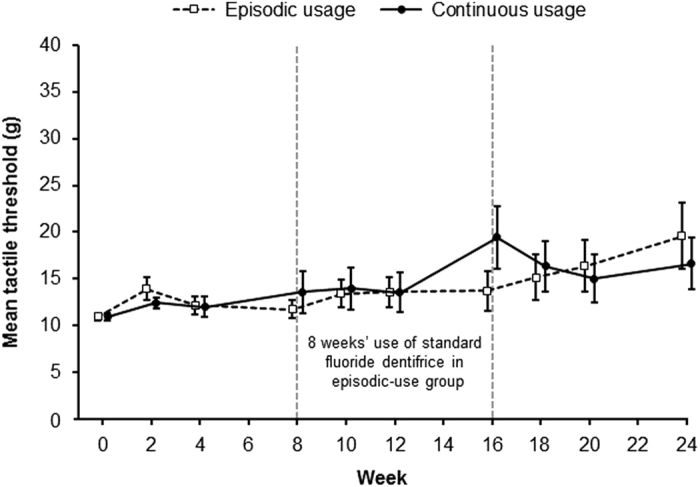
Mean (±s.e.) tactile threshold during continuous and episodic use of a desensitising dentifrice containing 5% calcium sodium phosphosilicate (intent-to-treat population). Tactile-threshold range 0–80 g. Data have been offset for clarity.

Small reductions were observed in mean tactile sensitivity, based on the tactile threshold scores, over the 24-week study period (see Figure 3 for mean scores (±s.e.) and Table 2 for adjusted mean change from baseline including 95% CIs). The profiles for the continuous- and episodic-use regimens were similar. Owing to the non-normal distribution of the data, median values were used to assess the change from baseline at weeks 8, 16 and 24 (Table 2); no statistically significant changes from baseline or differences between regimens were demonstrated at weeks 8, 16 or 24.

The profiles of the mean LMS scores for ‘intensity’, ‘duration’, ‘tolerability’ and ‘description’ were also similar for both regimens. Statistically significant (*P*<0.05) improvements from baseline to weeks 8, 16 and 24 were demonstrated for all LMS parameters except ‘duration’ scores at week 8 for the continuous-use group. There were no statistically significant between-treatment differences for change from baseline in LMS scores.

Similar profiles were also demonstrated for mean DHEQ endpoints for the continuous- and episodic-use groups, with little or no reduction in subject-perceived sensitivity over time. The two groups showed comparable profiles for raw TSQ scores, with little change over time in subject-reported sensitivity for either treatment regimen over the 24-week study period. The two treatment regimens demonstrated similar profiles with regard to the number of daily acidic challenges.

### Safety

A total of 23 treatment-emergent AEs were reported by 14 subjects (36.8%) in the episodic-use group (including two oral events in two subjects) and 36 treatment-emergent AEs were reported by 19 subjects (50.0%) in the continuous-use group (including 10 oral events in 10 subjects). None of the AEs were considered by the examiner to be treatment related. Two serious AEs were reported: one subject in the episodic-use group had severe concussion leading to withdrawal from the study; one subject in the continuous-use group reported bruised ribs. All AEs were of mild intensity with the exception of the severe concussion and a moderate laceration, experienced by the same subject.

## Discussion

This study demonstrated statistically significant improvements from baseline in dentinal hypersensitivity, as determined by Schiff sensitivity scores, with both episodic and continuous usage regimens of a 5% CSPS-containing desensitising dentifrice over a 24-week period. However, in this study no significant changes from baseline in tactile sensitivity were observed for either regimen. There were no significant between-regimen differences revealed by assessment of evaporative or tactile sensitivity. Similarly, subject-assessed endpoints demonstrated no difference between the two regimens. The study product was well tolerated when used continuously or episodically for 24 weeks.

Desensitising dentifrices are likely to be used intermittently in practice; however, very few published studies have attempted to investigate the consequences of episodic compared with continuous long-term usage of desensitising products. Studies by Jeandot *et al.*^27^ and Leight *et al.*^28^ demonstrated a return of sensitivity within 4 weeks of discontinuing potassium-containing dentifrices after 8 weeks of treatment. In addition, a comparison of dentifrices containing 5% CSPS and 5% potassium nitrate showed that both reduced sensitivity after 3 weeks’ treatment.^23^ Sensitivity started to increase again within 3 weeks of stopping treatment but to a greater extent following use of the dentifrice containing 5% potassium nitrate than the 5% CSPS-containing dentifrice.

Based on these studies, it was hypothesised that a return of sensitivity would be observed during a period of use of a standard dentifrice following regular use of a dentifrice containing 5% CSPS. However, owing to the lack of clinical evidence, the timing and degree of regression were unknown. The current study was therefore designed to explore these aspects of episodic use of a desensitising dentifrice. In contrast to standard efficacy studies,^35^ this study incorporated a parallel active-control arm for comparison of sensitivity during the off-treatment phase. Subjects who stopped active treatment were also monitored after recommencing active treatment to provide information on intermittent use.

Unexpectedly, this study did not demonstrate a difference in sensitivity between the episodic and continuous treatment regimens. One likely reason is the small improvement from baseline observed for both examiner-based measures of sensitivity with active treatment. These changes were inconsistent with previous studies of 5% CSPS dentifrices.^19,25^ For example, Sufi *et al.*^25^ reported an adjusted mean change from baseline of −0.80 (95% CI −1.05, −0.56) for Schiff sensitivity score and a median change of 5 (range 0–80) g for tactile threshold (Yeaple probe) after 8 weeks of treatment with a 5% CSPS-containing dentifrice. In comparison, the adjusted mean change from baseline in Schiff sensitivity score reported in the current study at 8 weeks was −0.36 and the median change from baseline in tactile threshold was 0.9 g. This may have confounded any impact on the overall findings of this study, making it difficult to demonstrate a statistically significant difference in sensitivity between regimens during the 8-week, off-treatment phase. The reasons behind the relatively small change in sensitivity from baseline in the current study are, however, unclear.

Another possible reason for the lack of detectable difference between the regimens is that an 8-week duration of withdrawal of 5% CSPS treatment was insufficient to demonstrate a measurable return of sensitivity, i.e., the protective effect of the occlusive calcium phosphate hydroxycarbonate apatite-like layer formed over the exposed dentine from the reaction when CSPS particles come into contact with the aqueous environment of saliva^7,13,14^ was not diminished. Future trials of episodic use may need to incorporate off-treatment periods of different lengths as well as other types of episodic regimens in order to provide guidance for dental professionals and patients on optimal use of desensitising dentifrices.

Twice-daily use of the 5% CSPS-containing dentifrice was generally well tolerated when used continuously for up to 24 weeks and almost all AEs were of mild intensity. No AEs considered to be related to the treatment were reported. This AE profile was consistent with that reported in studies of up to 8 weeks’ duration.^16,17,18, 19,20,21, 22,23,24, 25^

In conclusion, this exploratory study has demonstrated that twice-daily brushing with a 5% CSPS-containing desensitising dentifrice significantly improves dentinal hypersensitivity with either episodic or continuous long-term use and is generally well tolerated. However, the study did not demonstrate a difference in sensitivity control between the continuous and episodic regimens. Further studies incorporating different design elements may be necessary to provide information on intermittent use of desensitising dentifrices.

## Figures and Tables

**Table 1:** Demographic and baseline characteristics (safety population)

	*Episodic use (*n*=38)*	*Continuous use (*n*=38)*
*Sex,* n *(%)*		
Male	15 (39.5)	17 (44.7)
Female	23 (60.5)	21 (55.3)
		
*Age, years*		
Mean	27.8	31.9
Range	19–48	18–48
		
*Race,* n *(%)*		
Black or African American	0	1 (2.6)
White	38 (100)	37 (97.4)
		
*Stratification (by maximum baseline Schiff sensitivity score),* n *(%)*		
2	20 (52.6)	20 (52.6)
3	18 (47.4)	18 (47.4)

Abbreviation: CSPS, calcium sodium phosphosilicate.

**Table 2:** Change from baseline to weeks 8, 16 and 24 for Schiff sensitivity score and tactile threshold (intent-to-treat population)

	*Episodic use (*n*=38)*	*Continuous use (*n*=38)*	*Continuous versus episodic use*
*Schiff sensitivity score*			
Baseline mean (s.e.)	2.37 (0.07)	2.42 (0.08)	—
			
	*Change from baseline, adjusted mean (95% CI)*		*Difference (95% CI), P-value* a
Week 8	−0.36 (−0.52, −0.21)*	−0.26 (−0.42, −0.11)*	0.1 (−0.12, 0.32), 0.358
Week 16	−0.24 (−0.39, −0.09)*	−0.39 (−0.55, −0.24)*	−0.15 (−0.37, 0.07), 0.170
Week 24	−0.48 (−0.67, −0.29)*	−0.47 (−0.66, −0.29)*	0.01 (−0.25, 0.27), 0.941
			
*Tactile threshold (g)*			
Baseline mean (s.e.) (median)	10.92 (0.370) (10.00)	10.92 (0.370) (10.00)	—
			
	*Change from baseline, mean (±s.e.) (median)*		*Difference (95% CI), P-value* b
Week 8	0.71 (0.965) (0.00)	2.57 (2.313) (0.00)	0 (0.00, 0.00), 0.857
Week 16	2.65 (2.103) (0.00)	8.38 (3.382) (0.00)	0 (0.00, 0.00), 0.253
Week 24	8.48 (3.485) (0.00)	5.59 (2.791) (0.00)	0 (0.00, 0.00), 0.563

Abbreviations: CI, confidence interval; s.e., standard error.

a ANCOVA with fixed effects for treatment, visit and treatment-by-visit interaction; baseline Schiff score as covariate.

b CI for median difference based on Hodges–Lehmann method, *P*-value based on Wilcoxon rank-sum test.

*P<0.05, *t* test.
